# Supplementary figures and images for: Induction of SARS-CoV-2 Protein S-Specific CD8+ T Cells in the Lungs of gp96-Ig-S Vaccinated Mice
Source: Front Immunol. 2021 Jan 26;11:602254. doi: 10.3389/fimmu.2020.602254 (PMC7873992; doi:10.3389/fimmu.2020.602254)

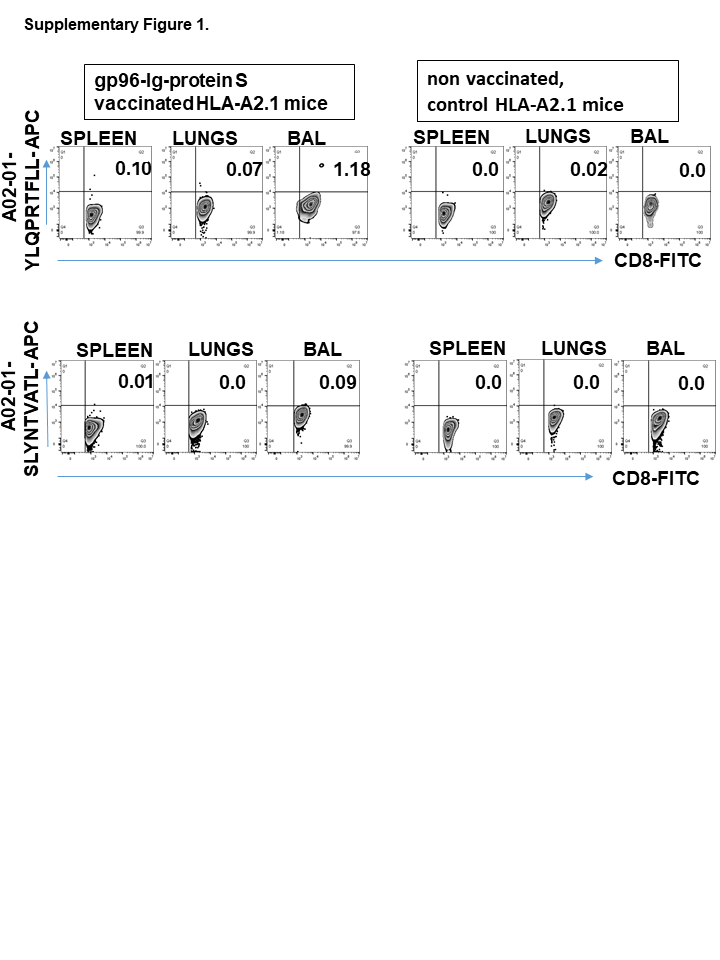

Supplement: Supplementary Figure 1 — Secreted Gp96-Ig-S vaccine induces S1-specific CD8 + cells in the spleen, lung tissue, and BAL. Thirty days after the vaccination of HLA-A2 transgenic mice, splenocytes, and lung cells and BAL were isolated from vaccinated and control mice (PBS). Cells were stained with HLA-A2 02-01 pentamer containing SARS-CoV2 YLQPRTFLL peptide, followed by surface staining for CD45, CD3, CD4, CD8, CD69, CXCR6. Representative zebra plots of gated CD8+ T cells expressing indicated pentamer-specific TCR+ CD8+ T cells in vaccinated and non-vaccinated HLA-A2 mice at day 30. HLA-A2 pentamer containing HIV/SIV (HIV-1 gag) SLYNTVATL pentamer was used as a negative control pentamer. [file Image_1.tif]
